# Sex Differences in Adult Autism Screening: A Comparison of Current Self-Report and Retrospective Parent-Report Measures

**DOI:** 10.1007/s10803-025-06753-8

**Published:** 2025-03-01

**Authors:** Michael Terner, Ofer Golan

**Affiliations:** 1 Department of Psychology, Bar-Ilan University, Ramat- Gan, 5290002 Israel; 2 OTI, The Israeli Autism Association, Giv’at-Shmuel, Israel

**Keywords:** Autism diagnosis, Autistic traits, Self-report, Parent-report, Developmental history, Sex differences

## Abstract

This study investigated sex differences in adult autism screening by comparing self-reports on current traits (Autism Spectrum Quotient; AQ) and parent-reports on childhood traits (Relatives Questionnaire; RQ). The aim was to examine the differential contribution of these distinct measures to diagnostic classification in both sexes. The study compared 102 clinically diagnosed autistic adults (30 females) and 152 non-autistic adults (60 females), aged 17–35 years. Participants completed the AQ, while their parents completed the RQ. Multivariate analysis of variance and sex-stratified discriminant analyses were employed to evaluate measurement patterns in males and in females. Significant main effects were found for diagnostic group on both measures and for sex on the RQ only. Group × sex interactions were significant for both measures. Within the autistic group, males and females showed no significant difference in AQ scores, but females scored significantly lower than males on the RQ. Discriminant analyses revealed high classification accuracies for both males (95.1%) and females (96.7%), with different weighting patterns between males (AQ = 0.597 [CI: 0.413–0.720], RQ = 0.712 [CI: 0.553–0.789]) and females (AQ = 0.763 [CI: 0.637–0.898], RQ = 0.478 [CI: 0.191–0.616]). The findings suggest that current self-report may be more central for identifying autism in females, while a more balanced combination of current-self and past-parent reports may be optimal for males. These sex-specific patterns highlight the importance of considering both current self-reported traits and developmental history in adult autism screening, with potential implications for improving diagnostic accuracy across sexes.

## Introduction

Autism is a life-long neurodevelopmental condition characterized by impairments in social communication, repetitive behaviors, restricted interests, and altered sensory sensitivities (American Psychiatric Association, 2022). Symptom expression and functional levels of autism are significantly heterogeneous, including highly variable cognitive and language abilities (American Psychiatric Association, 2022). Currently, 1 in 36 eight-year-olds in the USA is diagnosed with autism. Over the years, an increased estimated prevalence of autism has been reported (Maenner et al., 2023). Estimated rates of autism in adulthood range between 1.1% in the UK and 2.7% in the U.S. (Brugha et al., 2016; Maenner et al., 2023). In Israel, reports show an autism prevalence of 1.56% among native 8-year-olds (Dinstein et al., 2024).

Despite changes in recent years, autism is still more common in males than in females. The overall male-to-female prevalence ratio is estimated as 3.8:1, with autism prevalence of 43.0:1000 among boys and 11.4:1000 among girls (Maenner et al., 2023).

### The Importance of Developmental History for Adult Autism Diagnosis

As a neurodevelopmental condition, the diagnosis of autism necessitates evidence of autistic features throughout development, even when diagnosis is made in adulthood (American Psychiatric Association, 2022). These features must have been present from childhood, even if they were not recognized until social demands exceed one’s limited resources. However, obtaining this early developmental history can be challenging when diagnosing adults. Adults may remember their own perspective on their childhood but may find it harder to report on early experiences, or to reflect on the way they were perceived by their social environment, which is a requirement when examining diagnostic features such as socio-emotional reciprocity or non-verbal communication. Hence, even in adult diagnosis, parents or other relatives may be requested to report on their experiences with the diagnosed adult, during his childhood.

### Self-Identification of Autism and Screening in Adults

In contrast to pediatric assessments, where parent or caregiver reports often play a significant role, the identification and diagnosis of autism in adults emphasize self-reporting. This shift acknowledges the importance of individuals’ autonomy, lived experiences, and self-perceptions in health care settings (Overton et al., 2023). Compared to others’ reports, adults may better understand and communicate their autism-related experiences, feelings, and challenges (Lilley et al., 2022). Therefore, self-reporting is a fundamental pillar in adult screening processes, fostering a more nuanced, empathetic, and arguably effective approach. Indeed, research has shown that when reporting about camouflaging or about sensory experiences; self-report may be more representative than parent report. For example, Keith et al. (2019) found that adolescents reported higher levels of anxiety and auditory sensory symptoms than their parents. Moreover, adolescents’ self-reports were better correlated with autonomic measures of anxiety and sensory dysregulation. It seems that parent-report may miss the individual’s unique perspective on internal experiences (Keith et al., 2019). Therefore, relying on self-identification may not only foster an environment of trust and respect between healthcare professionals and adults seeking a diagnosis, but may also improve the diagnostic process itself (Overton et al., 2023).

### Sex^1^ Differences in Autism and Autistic Traits

Sex differences in autism and autistic traits have been a subject of extensive research. In the last decade, research has acknowledged that clinicians may have failed to recognize the unique profile of autistic women, especially those without intellectual disability. In addition to the underrepresentation of females in this group, females have been diagnosed at an older age, on average, compared to males (Fusar-Poli et al., 2022) and have been frequently misdiagnosed with various psychiatric conditions (Dell’Osso & Carpita, 2022). Additionally, parents may under-report the manifestation of autistic traits in females, as their first concerns of autism have been shown to differ by the child’s sex (Hiller et al., 2016; Little et al., 2017). In adulthood, a study by Taylor et al. (2022) found that females often self-reported more autistic traits than what was recognized by informants.

Research has also shown that females are more likely to camouflage or disguise their autistic characteristics (Lai et al., 2017; Mandy, 2019), which may have influenced the construction of autism criteria based predominantly on male behavior (Lai et al., 2017). Their tendency to camouflage, along with the potential under-recognition of their autistic traits by informants, suggest self-reporting may be more predictive of adult autism diagnosis in females, compared to males.

### Tools for Characterizing Current and Past Autistic Traits in Adults

As discussed earlier, diagnosing autism in adults requires an integration of information about both the current manifestation of autistic traits and relevant developmental history. One example for an instrument that addresses both past and present characteristics is the Social Communication Questionnaire (SCQ; (Rutter et al., 2003), which has two versions that focus on different temporal aspects. The Current version of the SCQ asks about current behaviors, while its Lifetime version considers the individual’s entire developmental history. However, the SCQ only sources information from parents or caregivers, and does not include self-report, which could limit its scope in capturing the adult’s current experiences.

Several instruments have offered the opportunity to compare self and other’s report on the current picture of adults’ autistic traits for example, the Social Responsiveness Scale-2 (SRS-2 Constantino & Gruber, 2012) focuses on the current presentation of autism traits, as reported by parents or caregivers and (in adults) by the individual himself. However, the SRS-2 focuses only on current presentation and fails to address changes throughout development, e.g., as a result of improved ability to camouflage. This approach may result in an incomplete understanding of the assessed individual’s life-long experience.

Two widely used tools for the purpose of autism screening in adults are the Autism Spectrum Quotient (AQ) and the Relatives’ Questionnaire (RQ). The AQ allows adults to self- report on current autistic features, while the RQ allows a parent or a caregiver to provide information about relevant features in childhood.

The AQ, developed by Baron-Cohen and colleagues in 2001, is a self-administered instrument divided into five key components: social skills, communication, attention to detail, attention switching, and imagination. It was designed to detect individual variations in autistic traits and serves as a preliminary screening tool (Baron-Cohen et al., 2001; Woodbury-Smith et al., 2005). Recent meta-analytic findings by Barańczuk et al. (2024) revealed a nuanced pattern of sex differences in autistic traits as measured by the AQ. While males exhibited significantly elevated autistic traits in non-clinical populations, this effect was not observed in autistic populations. These findings extend previous general population-level research (Ruzich et al., 2015).

The RQ, adapted from the Childhood Autism Spectrum Test (CAST; Scott et al., 2002), comprises items related to social and communication impairments, as well as repetitive or stereotypic behavior. These questions are answered by a relative, preferably a parent, who reflects retrospectively on the individual’s childhood. The foundational CAST instrument has been validated as an effective screening method for identifying more subtle manifestations of autism among children aged 4–11 years (Baron-Cohen et al., 2009). The CAST has been adapted and translated into over twenty-two languages. Its psychometric properties have been examined in various cultures and languages, including Spanish (Morales-Hidalgo et al., 2017), Mandarin (Sun et al., 2014), Bulgarian (Vulchanova, 2016), and Brazilian (Ribeiro et al., 2022), confirming its widespread application and cross-cultural relevance. The CAST consistently reveals significant sex-based variations in autism trait identification, with male participants demonstrating higher scores across diverse cultural and linguistic adaptations, including British (Williams et al., 2008), Spanish (Morales-Hidalgo et al., 2017), Mandarin (Sun et al., 2014 and Hebrew (Terner et al., 2024). Existing studies on the use of the CAST in its RQ form (i.e., retrospective reports by adults’ parents) have presented mixed results, suggesting a need for further empirical exploration to fully understand its efficacy in this context (Jones et al., 2023; Kenny & Stansfield, 2016).

### The Current Study

This study examined two complementary screening approaches in adult autism identification: self-reported current traits (Autism Spectrum Quotient; AQ) and informant-reported childhood traits (Relatives Questionnaire; RQ). We investigated the differential contribution of these temporally distinct measures to diagnostic classification, with particular emphasis on sex-specific diagnostic utility. The investigation employed both multivariate analysis of variance and sex-stratified discriminant analysis to comprehensively evaluate measurement patterns in both males and females.

Based on the above literature, the following hypotheses were formulated:

1. Autistic individuals will show higher scores than non-autistic individuals both on current self-report (AQ) and on retrospectively parent-report (RQ) measures.
2. Males will show higher scores than females on current self-reported and on retrospective parent reported measures.
3. An interaction effect between diagnostic group and sex on current self-report scores was hypothesized. Following Barańczuk et al. (2024), we predicted that sex differences (with males scoring higher than females) will be greater for the non-autistic, compared to the autistic group.
4. An interaction effect between diagnostic group and sex was also hypothesized for retrospectively reported childhood scores. Due to parents’ difficulties to recognize autism traits in girls (Taylor et al., 2022), we predicted that sex differences (with males scoring higher than females) will be greater for the autistic, compared to the non-autistic group.
5. Given documented sex differences in autism presentation and recognition, distinct discriminant functions were expected in males and females.

## Method

### Participants and Procedure

AQ, RQ and demographic data of the autistic group were retrieved from two clinical centers, operated by OTI – the Israeli Association for Autism. These two tertiary centers provide diagnostic assessment services for autistic individuals. A waiver of consent for this retrospective records review was given by the Ness-Ziona Beer-Yaacov Mental Health Center’s Helsinki Committee (#543). The record review yielded data on 102 clinically diagnosed autistic adults (30 assigned female at birth) aged 17.5–35 (M = 24.03, SD = 5.05). To be included, participants had to have a clinical diagnosis of autism given by a psychiatrist or a clinical psychologist according to DSM-5 criteria, with no comorbid intellectual impairment.

Of the autistic group, 41% of the cases attended special education as children. 70% had previous neurodevelopmental diagnoses (most commonly, ADHD, learning difficulties), 41% had a previous PDD/autism diagnosis, and 43% had previous psychiatric diagnoses (most commonly anxiety, depression, OCD), whereas 2% had sensory/genetic/other medical conditions.

The non-autistic sample consisted of 152 participants (60 assigned female at birth), aged 18–35 (M = 23.55, SD = 3.14). Most were single (63.15%) and had a high school graduation educational level (59.21%). 9.21% reported having a neurodevelopmental diagnosis (mostly ADHD) and 7.24% reported having a psychiatric diagnosis (mostly anxiety).

Non-autistic participants were recruited from the community through undergraduate research assistants. Inclusion criteria ensured no self-reported diagnosis of autism, an AQ score that does not exceed the general population cutoff of 30 (Golan et al., 2022) and no reported severe mental illness. All non-autistic participants provided informed consent. Ethical approval was obtained from the authors’ Departmental ethics committee.

Using the Qualtrics XM platform, non-autistic participants completed the AQ, and their parents filled out the RQ.

The autistic and non-autistic groups were comparable on age (*t*[153.40]=-0.86, N.S.) and sex (*χ*^2^[1] = 2.70, N.S.).

### Measures

*The Autism Spectrum Quotient (AQ;* Baron-Cohen et al., 2001) is a 50 item self-report instrument measuring autistic traits. Participants rate their agreement with each statement on a 4-point Likert-type scale, ranging from “definitely agree” to “definitely disagree.” Participants’ ratings on each item are then collapsed to binary scores (agree/disagree) with the autistic trait response scoring 1. The total score range is therefore 0–50. The AQ was created to identify variations in autistic traits and has also been used as a screener for autism diagnosis in adults. Diagnostic data values showed a diverse range, with accuracy values spanning from 0.72 to 0.90, the area under the curve (AUC) ranging from 0.65 to 0.99, sensitivity between 0.75 and 0.95, and specificity from 0.52 to 0.97 (Baghdadli et al., 2017). The AQ has been translated into 36 languages, including a Hebrew version (Golan et al., 2022). The Hebrew version of the AQ demonstrated strong internal consistency (Kuder-Richardson 20 = 0.90) and a notable AUC of 0.94. Employing a cutoff of 21 for clinically referred adults, the tool showed a sensitivity of 0.90, a specificity of 0.76, a positive predictive value (PPV) of 0.90, and a negative predictive value (NPV) of 0.77 (Golan et al., 2022).

*The Relatives Questionnaire (RQ) * is an adaptation of the Childhood Autism Spectrum Test (*CAST*; Scott et al., 2002), asking an adult’s relative to retrospectively report on the adult’s autistic traits as a child. The instrument employs binary responses (yes/no), and scores range from 0 to 31. Test-retest reliability of the original CAST was found to be 0.83 (Williams et al., 2006) with sensitivity of 1, specificity of 0.97, and a positive predictive value of 0.50 at the designated cut-point of 15 (Williams et al., 2006). The CAST and its RQ version were translated by the last author in collaboration with the original instrument’s creator. Terner et al. (2024) examined the Hebrew version of the CAST (CAST-Heb) employing an optimal cutoff score of 9, that was particularly more suitable for boys than girls. In two separate studies, the CAST in its adult RQ form has shown sensitivity of 0.77–0.79 and specificity of 0.45–0.50. for the original cutoff of 15, with PPV ranging between 0.41 and 0.88, NPV ranging between 0.30 and 0.82 and AUC ranging between 0.67 and 0.71 (Jones et al., 2023; Kenny & Stansfield, 2016).

To evaluate the psychometric properties of the RQ for screening autism in an Israeli sample, a Receiver Operating Characteristic (ROC) analysis was conducted. Its results revealed an Area Under the Curve (AUC) of 0.959, 95% CI [0.935, 0.982], indicating excellent discriminative ability between autistic and non-autistic adults. This AUC value notably exceeds previously reported ranges of 0.67-0.71 for the adult RQ (Jones et al., 2023; Kenny & Stansfield, 2016).

At a cutoff score of 9 (comparable to the children’s CAST-Heb), the RQ demonstrated high sensitivity (0.89) and specificity (0.88). Since PPV and NPV should be calcuated, in relation to the prevalence in the examined population, the porportion of the adults receiving an autism diagnosis out of those applying for an autism diagnosis in the tertiary clinical centers included was examined and found to be 70%. Based on this prevalence, PPV was 0.95, NPV was 0.78, and the overall accuracy of the RQ in this study was 0.89.

## Results

A two-way MANOVA examining the effects of group and sex on AQ and RQ scores revealed significant multivariate main effects for group (Wilks’ λ = 0.266, F(2, 249) = 344.120, *p* <.001, η²ₚ = 0.734), sex (Wilks’ λ = 0.912, F(2, 249) = 11.992, *p* <.001, η²ₚ = 0.088), and a significant group × sex interaction (Wilks’ λ = 0.951, F(2, 249) = 6.348, *p* =.002, η²ₚ = 0.049).

Univariate analyses showed significant main effects of group for both AQ (F(1, 250) = 444.603, *p* <.001, η²ₚ = 0.640) and RQ (F(1, 250) = 375.992, *p* <.001, η²ₚ = 0.601). The sex main effect was significant for RQ (F(1, 250) = 21.610, *p* <.001, η²ₚ = 0.080) but not for AQ (F(1, 250) = 0.445, *p* =.505, η²ₚ = 0.002).

**Fig. 1:**
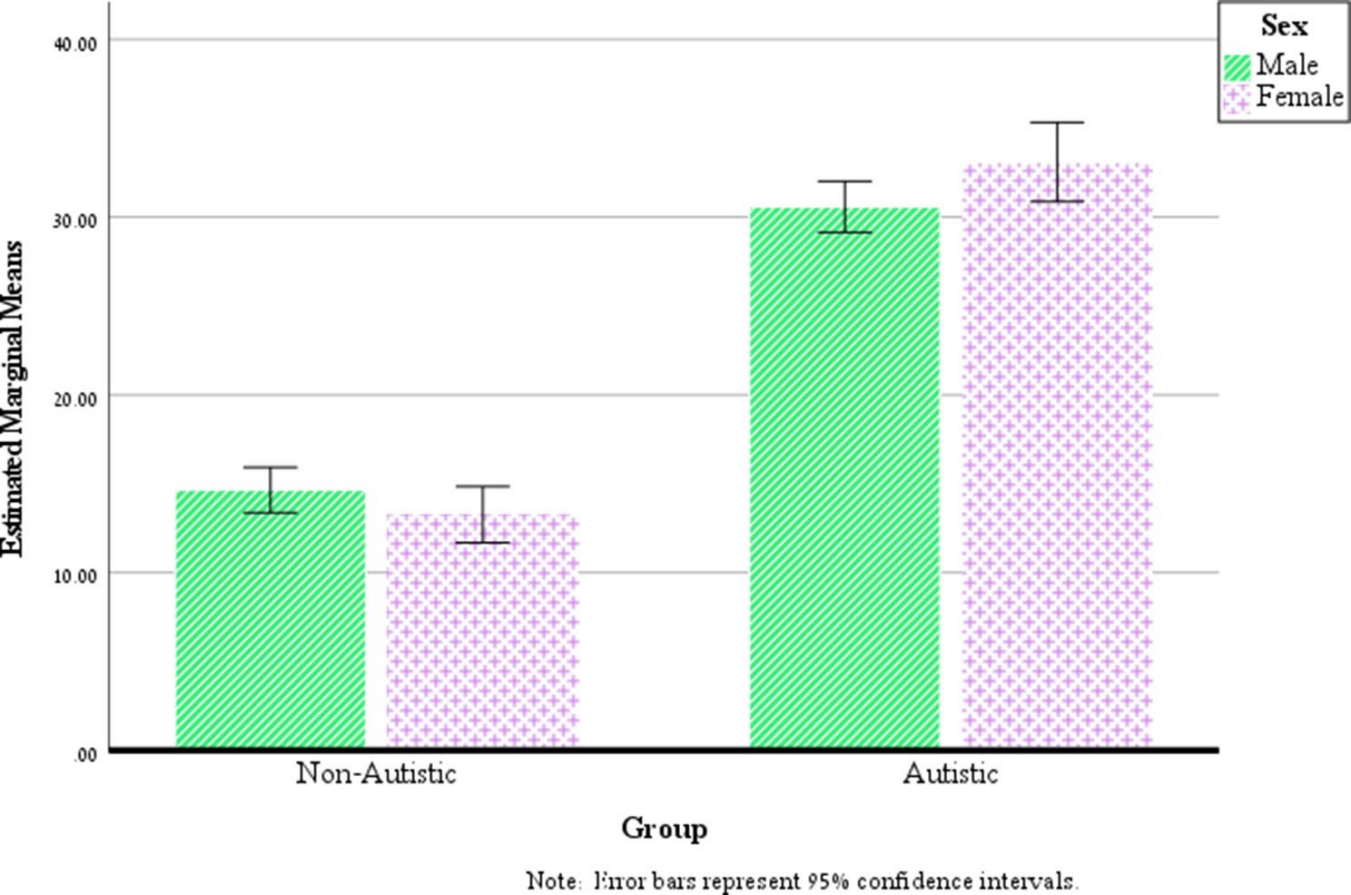
Estimated marginal means of AQ scores comparing autistic and non-autistic groups by sex

The group × sex interaction was significant for AQ scores (F(1, 250) = 5.293, *p* =.022, η²ₚ = 0.021). Simple main effect analysis revealed no significant difference within the autistic group between males (M = 30.58, SE = 0.731, 95% CI [29.143, 32.023]) and females (M = 33.10, SE = 1.133, 95% CI [30.869, 35.331]; *p* =.063), with overlapping confidence intervals indicating a trend toward higher scores in females. There were also no significant sex differences within the non-autistic group (males: M = 14.65, SE = 0.647, 95% CI [13.378, 15.926]; females: M = 13.27, SE = 0.801, 95% CI [11.689, 14.844]; *p* =.180). These results are illustrated in Fig. 1.

**Fig. 2:**
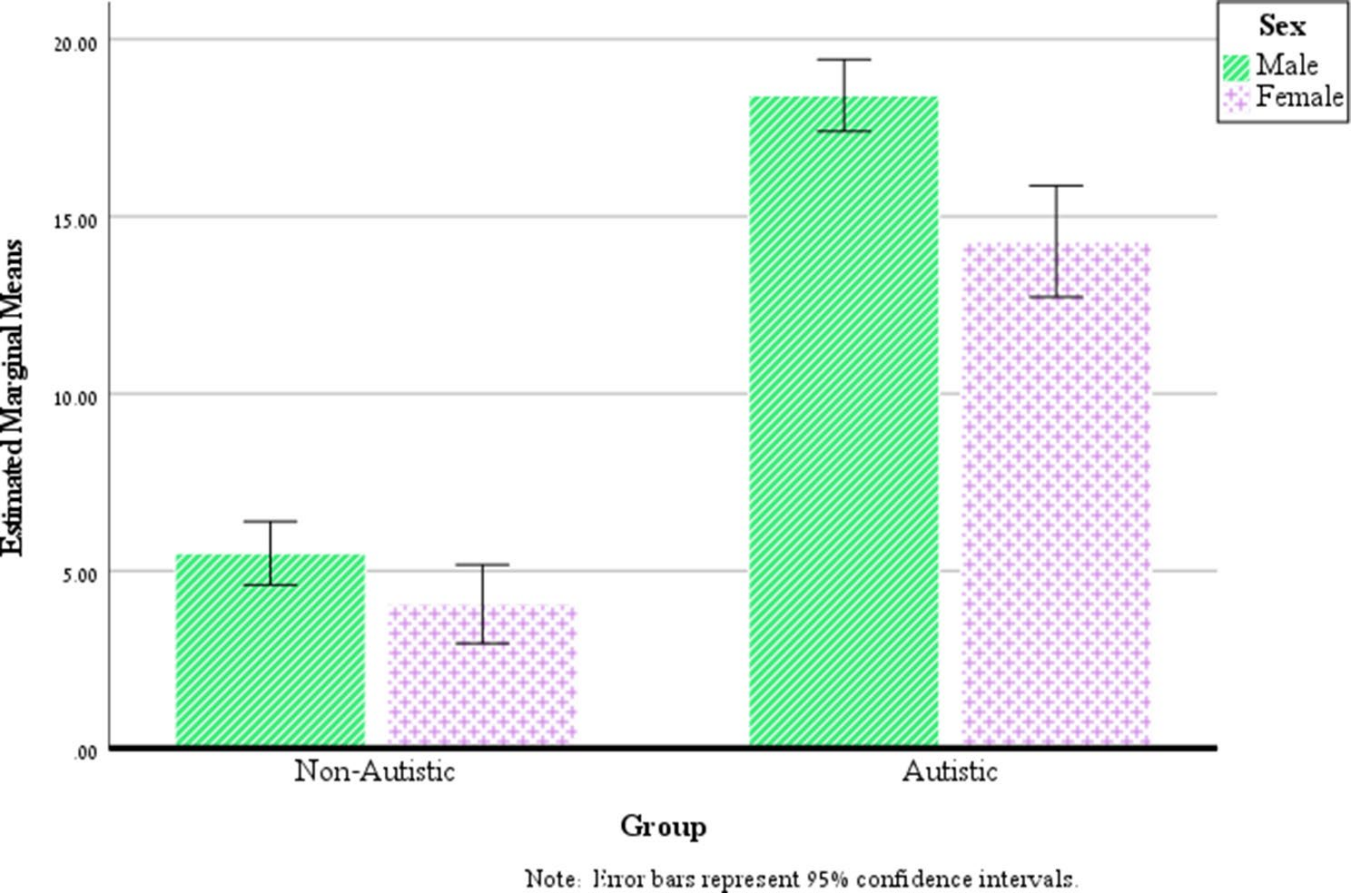
Estimated marginal means of RQ scores comparing autistic and non-autistic groups by sex

The group × sex interaction was also significant for RQ scores (F(1, 250) = 5.052, *p* = .025, η²ₚ = .020). Simple main effect analysis revealed that within the autistic group, females (M = 14.30, SE = 0.797, 95% CI [12.730, 15.870]; *p* <.001) scored significantly lower than males (M = 18.42, SE = 0.515, 95% CI [17.403, 19.430]), representing the only instance where confidence intervals showed no overlap. For RQ scores in the non-autistic group, while males scored higher (M = 5.50, SE = 0.455, 95% CI [4.603, 6.397]) than females (M = 4.07, SE = 0.564, 95% CI [2.956, 5.177]; *p* =.049), the confidence intervals showed a substantial overlap. These results are illustrated in Fig. 2.

Sex-stratified discriminant analyses revealed high classification accuracies for both males (95.1%; canonical correlation = 0.864, Wilks’ λ = 0.254, *p* <.001) and females (96.7%; canonical correlation = 0.886, Wilks’ λ = 0.215, *p* <.001). The standardized coefficients suggested differential weighting patterns in the two sex groups: Whereas in males the RQ weighed more strongly than the AQ (AQ = 0.597, RQ = 0.712), in females, the opposite direction was found (AQ = 0.763, RQ = 0.478). To assess the statistical significance of the differential weighting patterns, we computed 95% confidence intervals for the standardized canonical discriminant function coefficients using a double bootstrap procedure. For females, the confidence intervals were non-overlapping, with current self-report (AQ: CI [0.637–0.898]) showing significantly higher weights than retrospective parent-report (RQ: CI [0.191–0.616]). In males, the confidence intervals showed substantial overlap (AQ: CI [0.413–0.72], RQ: CI [0.553–0.789]), suggesting more balanced contributions from both measures.

## Discussion

The present study examined the comparative utility of current self reports and parental reports on childhood in adult autism identification, with a specific focus on sex differences in the manifestation of autistic traits. Consistent with our first hypothesis, autistic individuals scored significantly higher than non-autistic individuals on both the AQ (current self report) and the RQ (parental report about childhood). These results reinforce the utility of both measures in distinguishing between autistic and non-autistic adults. The strong main effects for diagnostic group on both measures align with prior research demonstrating the utility of the AQ in detecting self-reported autistic traits (Baghdadli et al., 2017) and of the RQ in capturing parent-reported retrospective childhood features of autism (Kenny & Stansfield, 2016). In line with our second hypothesis, males scored higher than females on the RQ, reflecting informant-reported childhood traits. This result resonates similar findings of sex differences reported by children’s parents on the CAST (Williams et al., 2008; Terner et al., 2024). Our finding of no significant sex differences in AQ scores runs contrary to some literature demonstrating higher AQ scores in males, compared to females (Ruzich et al., 2015; Barańczuk et al., 2024). We examined potential reasons for this gap in the next hypothesis, looking into sex differences in autistic and non-autistic adults. Our third hypothesis predicted greater sex differences (with males scoring higher than females) in the non-autistic compared to the autistic group. This hypothesis was partially supported by the data. The significant interaction effect between diagnostic group and sex for AQ scores revealed distinct patterns across diagnostic groups. Within the autistic group, we found no significant sex differences, aligning with the findings of Schuck et al. (2019), who similarly observed a marginally-significant trend towards higher scores in autistic females, compared to autistic males. In the non-autistic group, the difference between males and females did not reach significance. This finding diverges from the meta-analysis of Barańczuk et al. (2024), which demonstrated significantly higher AQ scores among non-autistic males. Our relatively modest sample size, may have limited our ability to detect such differences. It may have also contributed to our earlier reported non-significant main effect of sex on AQ scores. The interaction between diagnostic group and sex for RQ scores revealed a distinct pattern that supported our fourth hypothesis regarding retrospective parent-reported childhood features of autism. As predicted, sex differences were more pronounced within the autistic group, where females scored significantly lower than males. This was the only comparison where confidence intervals showed no overlap, suggesting a robust effect. In contrast, while non-autistic males scored higher than non-autistic females, the confidence intervals demonstrated substantial overlap, indicating a less pronounced difference. These findings align with previous research suggesting that parents may have greater difficulty recognizing autistic traits in females during childhood (Hiller et al., 2016), potentially due to differences in the presentation of autistic features or due to increased camouflaging behaviors among females (Lai et al., 2017). This pattern of results underscores the importance of considering sex-specific manifestations of autism when evaluating retrospective parent reports in diagnostic assessments. Our fifth hypothesis regarding sex-specific differences in classification accuracy was supported. Both self-reports and parental reports contributed significantly to the discrimination between autistic and non-autistic adults, achieving classification accuracies of 95.1% for males and 96.7% for females. These findings align with current understanding of autism as a complex, lifelong neurodevelopmental condition, the diagnosis of which relies both on current symptom manifestation and on developmental history (APA, 2022). Our findings revealed distinct sex-specific patterns in diagnostic indicators. For females, current self-report demonstrated significantly higher discriminant weights compared to retrospectively reported childhood traits, with non-overlapping confidence intervals indicating a robust statistical difference. In contrast, males showed more balanced contributions from both measures, with substantially overlapping confidence intervals. These findings highlight the value of incorporating multiple informant perspectives in diagnostic assessment. While both measures contribute meaningful diagnostic information across sexes, their relative contributions appear to differ between males and females. Valuable complementary information may also be achieved from other family members (e.g., siblings), educators, and healthcare providers (Kang et al., 2023), though the relative weighting of such additional perspectives requires further investigation. Several factors may account for this difference:

***Under-recognition of female autistic traits:*** Parents may find it more challenging to identify autistic features during childhood in females compared to males (Hiller et al., 2016). Autistic females may exhibit less overt symptoms, such as social communication difficulties and restricted interests, which are less noticeable to caregivers compared to the more prototypical presentation of autism in males (Wood-Downie et al., 2021a, b).

***Camouflaging***: Autistic females are known to camouflage their traits, masking their unique autistic features and behaving as “socially expected”. Following this behavior, self-reports may better capture nuanced or internalized aspects of autism that might be overlooked in parental or clinical observations (Hull et al., 2020; Jorgenson et al., 2020; Milner et al., 2023; Wood-Downie et al., 2021a, b).

***Self-Identification***: Overton et al. (2023) documented elevated female representation in self-identified autistic populations compared to formally diagnosed cohorts. This phenomenon may be attributed to enhanced trait recognition capabilities among autistic females, facilitating autonomous self-identification without parental corroboration. These findings align with established sex differences in social cognition and their implications for self-awareness in autism (Greenberg et al., 2023).

### Limitations and Future Research

While this study provides valuable insights, some limitations should be noted. First, the sample composition presents potential bias, comprising individuals already seeking diagnostic evaluation who may not represent autism screening patterns in the general population. Although diagnoses were based on standardized diagnostic tools, the non-independence of diagnostic status from informant reports represents a methodological challenge, as correlated measurement error could influence associations between reports and diagnostic outcomes.

Another measurement consideration involves the AQ and RQ instruments, which differ along both informant type (self vs. parent) and temporal focus (current vs. childhood). Future studies may wish to examine these factors systematically, i.e. to examine current, as well as childhood-focused, self and parental reports. Moreover, both the AQ and the RQ, while valuable in autism research, may not fully align with DSM-5 criteria for autism. This limitation underscores the need for either substantial adjustments to these tools or the development of new instruments, better aligned with current diagnostic guidelines (Smith et al., 2015). Our findings suggest such instruments should examine ASD characteristics according to its up to date formulation, and that variable presentation of traits in males and in females, currently and in childhood, should be taken into account.

Our findings are also limited by the absence of comprehensive participant characteristics from clinical records, including cognitive abilities, language skills, and socioeconomic status of both autistic individuals and parent filling out the RQ. These unmeasured variables could potentially influence assessment outcomes and questionnaire responses, limiting our ability to examine mediating or moderating effects.

Additionally, the study’s reliance on binary gender categories limits our understanding of autistic trait manifestation across diverse gender identities. This limitation points to the need for more gender-inclusive approaches in future research to fully capture differences in reporting across gender spectra.

Several directions for future research emerge from these limitations. Longitudinal studies evaluating the long-term consistency of self and parental reports could advance our understanding of whether reporting patterns remain stable or shift throughout development in autistic individuals. Furthermore, research with larger community samples is warranted to assess the generalizability of these self-report and parental-report patterns.

Cultural considerations represent another key area for future investigation. Cultural variables related to social/gender norms and stigma may significantly impact one’s willingness or ability to report autistic traits. Cross-cultural studies could offer valuable insights into developing optimal, culturally responsive approaches for incorporating self- and observer-reports in autism identification across diverse communities.

### Implications for Clinical Practice

The findings from this study offer several important implications for clinical practice in adult autism assessment. The differential patterns observed between self-report and parental-report measures across sex and diagnostic groups suggest the need for a more nuanced, multi-informant approach to autism screening process.

A key consideration involves the distinct temporal perspectives captured by the AQ and RQ instruments. While the AQ assesses current autistic traits through self-report, the RQ elicits retrospective parental observations of childhood characteristics. This temporal distinction necessitates careful interpretation of comparative analyses and highlights the importance of considering developmental trajectories in diagnostic formulation.

The sex-specific patterns in reporting measures warrant consideration in clinical settings. For clients assigned female at birth, our findings suggest that while both measures contribute to diagnostic classification, self-reported experiences may carry relatively greater weight than parental reports about childhood symptoms. This consideration becomes especially salient given the documented tendency of females to camouflage autistic traits (Hull et al., 2020; Milner et al., 2023) and the potential under recognition of autism characteristics in females by parents during childhood (Hiller et al., 2016).

For clients assigned male at birth, the more balanced contributions of both current and retrospective measures requires a relatively equal consideration of both perspectives in diagnostic formulation. This is particularly significant given that autistic males may demonstrate poorer self awareness, compared to autistic females, potentially impacting their ability to recognize and report their own social differences (Greenberg et al., 2023). Consequently, parental reports may offer crucial complementary information about behavioral patterns and developmental history for male clients.

The findings regarding measurement bias necessitate careful consideration in clinical settings. Practitioners should remain cognizant that existing screening tools may exhibit sex-specific biases that could lead to underestimation of autistic traits in the developmental history of females (Murray et al., 2017; Belcher et al., 2023). Moreover, the temporal distinction between current self-report and retrospective parental report introduces additional complexity in measurement interpretation, requiring careful consideration of potential recall bias and developmental changes in symptom presentation.

In conclusion, the current study provides empirical support for the combined use of self-reports and parental reports in the autism screening process of adults and underscores the importance of considering sex differences in this context. These findings have the potential to inform and enhance clinical practices, leading to more accurate and nuanced understandings of autism in adults. A more holistic approach, incorporating both self-report and parental report, is suggested to be essential for an accurate autism diagnosis in adults, respecting the self-perception and autonomy of individuals while also acknowledging the significance of developmental history in understanding their current condition.

## Footnotes

1 We Acknowledge that Gender Identity Differences are more Common in Autism (Overton et al., 2023). However, since we had no Access to Participants’ Gender data, the Terms ‘males’ and ‘females’ throughout this work, Relate to Participants’ Assigned at Birth sex.
